# Investigating the Potential Use of Environmental DNA (eDNA) for Genetic Monitoring of Marine Mammals

**DOI:** 10.1371/journal.pone.0041781

**Published:** 2012-08-29

**Authors:** Andrew D. Foote, Philip Francis Thomsen, Signe Sveegaard, Magnus Wahlberg, Jos Kielgast, Line A. Kyhn, Andreas B. Salling, Anders Galatius, Ludovic Orlando, M. Thomas P. Gilbert

**Affiliations:** 1 Centre for GeoGenetics, Natural History Museum of Denmark, University of Copenhagen, Copenhagen, Denmark; 2 Department of Bioscience, Aarhus University, Roskilde, Denmark; 3 Fjord&Bælt, Kerteminde, Denmark; 4 Marine Biological Laboratory, University of Southern Denmark, Kerteminde, Denmark; University of Connecticut, United States of America

## Abstract

The exploitation of non-invasive samples has been widely used in genetic monitoring of terrestrial species. In aquatic ecosystems, non-invasive samples such as feces, shed hair or skin, are less accessible. However, the use of environmental DNA (eDNA) has recently been shown to be an effective tool for genetic monitoring of species presence in freshwater ecosystems. Detecting species in the marine environment using eDNA potentially offers a greater challenge due to the greater dilution, amount of mixing and salinity compared with most freshwater ecosystems. To determine the potential use of eDNA for genetic monitoring we used specific primers that amplify short mitochondrial DNA sequences to detect the presence of a marine mammal, the harbor porpoise, *Phocoena phocoena*, in a controlled environment and in natural marine locations. The reliability of the genetic detections was investigated by comparing with detections of harbor porpoise echolocation clicks by static acoustic monitoring devices. While we were able to consistently genetically detect the target species under controlled conditions, the results from natural locations were less consistent and detection by eDNA was less successful than acoustic detections. However, at one site we detected long-finned pilot whale, *Globicephala melas*, a species rarely sighted in the Baltic. Therefore, with optimization aimed towards processing larger volumes of seawater this method has the potential to compliment current visual and acoustic methods of species detection of marine mammals.

## Introduction

The use of molecular genetic markers for monitoring biodiversity and detecting and identifying species, individuals or measuring population genetic parameters can provide valuable information for the management and conservation of species and ecosystems [1]. Non-invasive sampling using hair or scat has been successfully used in genetic monitoring programs of wide-ranging terrestrial or semi-aquatic species, which often occur at low density [1] and can be a lower cost approach than direct sampling as the individual or species does not have to be directly encountered and greater number of samples can be collected. However, this is offset by what can be increased DNA extraction and sequencing costs and also a decrease in returns to the potentially large numbers of duplicate samples [2]. For fully aquatic species such as cetaceans, the collection of non-invasive samples is less straightforward and often (but not always; see ref [3]) requires the sampler to directly encounter the target species to collect non-invasive samples such as feces, sloughed skin or exhalation blow [3]–[6]. Current non-invasive sampling of marine species therefore has many of the same logistical and financial costs as biopsy sampling. However, biological excretory processes such as the sloughing of skin, urination and defecation can be sources of ‘environmental’ DNA (eDNA), and can provide a record of the species' presence over the period that the DNA persists in the environment [7]–[14].

In freshwater aquatic environments the use of eDNA for genetic monitoring has been tested by a number of recent studies (e.g. [9], [11]–[14]), which suggest that eDNA is homogenously distributed within freshwater systems and can be effectively used to detect and even quantify species presence [11]. Here, we investigate whether eDNA from the water column can be used to detect target species occurrence of mammals in the marine environment. The use of seawater samples for eDNA analysis is likely to be more challenging than freshwater due to the larger body of source water, strong tide and current action, which will rapidly dilute and disperse the eDNA. Further, the high salinity of the samples may also render amplification of eDNA by polymerase chain reactions (PCRs) more prone to inhibition [15]. Despite these potential drawbacks, quantification of DNA in the sea has been used to provide an indicator of biomass in marine ecosystems [16], and sampling and sequencing of intracellular DNA of microorganisms sampled from seawater has enabled the metagenomic investigation of their biodiversity and community structure [17], [18].

In this study, we test the potential for using eDNA to detect the presence of marine mammals, by using as a model a small cetacean species, the harbor porpoise *Phocoena phocoena*. The harbor porpoise is the only regularly occurring cetacean species in the western Baltic, the region where this study took place, although the species is rare in the inner Baltic [19], [20]. Static acoustic monitoring devices which log detected echolocation click trains of harbor porpoises provide a record of occurrence and relative density at each site [21]–[23] and reliable field validation of our eDNA based tests. Here, we experimentally demonstrate, using both controlled conditions and natural populations, the feasibility of targeted eDNA based animal detection from seawater samples.

## Methods

**Figure 1:**
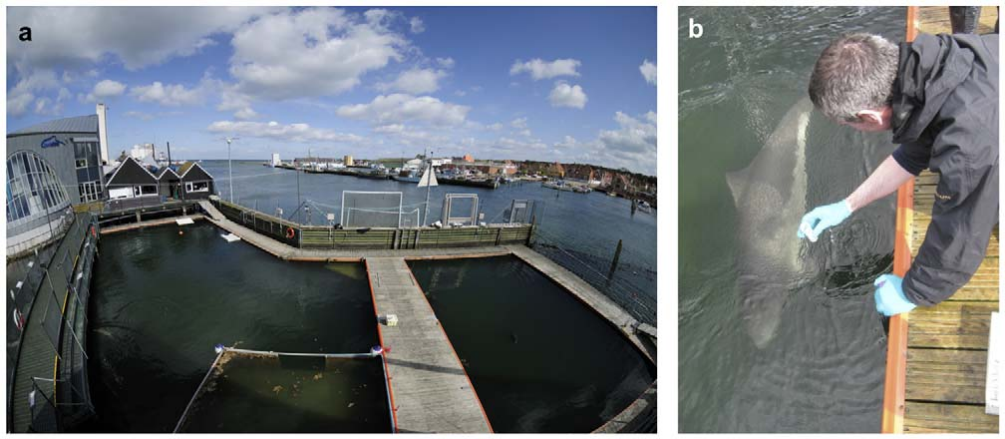
The sea pen at Fjord&Bælt in Kerteminde, Denmark. Figure 1a shows the sea pen containing approximately 4 million liters of seawater and 4 harbor porpoise. The two ends of the pen are comprised of netting, allowing tidal seawater to move through the pen (photograph © Solvin Zankl). Figure 1b shows the sampling of 15 ml of seawater from the sea pen (photograph © Ross Culloch).

Seawater samples were collected under both controlled conditions, and from natural field sites. The controlled site was a sea pen in a natural harbor basin at Fjord&Bælt (F&B) in Kerteminde, Denmark (Fig. 1a). The pen holds four harbor porpoise in approximately 4 million liters of seawater, which is flushed daily by the tidal water movements in the harbor basin, that enter at the netted ends of the pen. Five 15 ml water samples were collected at a depth of approximately 50 cm from different points around the perimeter of the sea pen in a sterile container, which was sealed until just prior to sampling and handled using unused sterile latex gloves, which were discarded after the collection of each sample (Fig. 1b). An additional 45 samples of 15 ml were collected at varying distances (∼0–1 km) from the pen in the direction of the ebbing tide. After collection, 1.5 ml of 3 M sodium acetate and 33 ml absolute ethanol was added to the water samples to precipitate any extracellular DNA (final concentrations 0.09 M, and 66% respectively), which were then stored at −20°C until DNA extraction. As a control, a small layer of epidermis was also collected from each of the four porpoises using Scotch tape. Epidermal samples were stored in 20% dimethyl sulphoxide (DMSO) saturated with NaCl at −20°C until DNA extraction.

Seawater samples were also collected during August 2011 at 8 locations in the western Baltic at sites where static acoustic monitoring devices, C-PODs (Cetacean and POrpoise Detector, Chelonia Ltd., U.K.), were deployed as part of the project Static Acoustic Monitoring of the Baltic Sea Harbor Porpoise (SAMBAH). The water samples were collected approximately 50 cm below the surface as above, employing the same protocols to reduce the risk of contamination. Three 50 ml samples were collected at each acoustic datalogger site and a 15 ml aliquot was taken from each of these samples and treated as above. The data recorded by the C-PODs were analyzed with the software CPOD.exe (v 2.021) with the filters ‘NBHF’ and ‘Other Cetaceans’ to search for porpoise clicks and clicks from other cetaceans, respectively. The percentage of porpoise positive days (i.e. days in which porpoise clicks were detected) were calculated for each site during the three month recording period prior to the date of water sampling (Table 1). No specific permits were required for the described field studies.

**Table 1:** Detection of harbor porpoise DNA using qPCR at a controlled site (Fjord&Belt pen) and at natural sites.

	Acoustic detection	Genetic detection	
Location	% Porpoise positive days	Positive PCRs	Cycle threshold
Positive control (DNA extracted from skin)		3/3	18, 18, 18
Fjord&Bælt pen		3/3	34, 35, 35
<10 m from F&B pen		1/3	49
>10 m from F&B pen		0/3	-
Site 1	94	1/3	49
Site 2	42	0/3	-
Site 3	63	0/3	-
Site 4	6	0/3	-
Site 5	0	0/3	-
Site 6	0	0/3	-
Site 7	0	2/3	38*, 50*
Site 8	79	0/3	-

Genetic detections at the eight natural sites are compared with acoustic detection rates based on data from static acoustic monitoring devices over the three months prior to eDNA sampling.

* sequencing of PCR clones indicates these were genetic detections of long-finned pilot whale and not genetic detection of harbor porpoise at this site.

DNA from seawater samples was extracted in a dedicated clean lab in a building separate from the location of post PCR work and extraction of DNA from the epidermis samples. Rigorous controls for preventing and monitoring contamination adopted from ancient DNA protocols were employed. Seawater samples were centrifuged at 6000 *g* for 10 min to pellet any precipitated DNA. One blank extraction using molecular biology grade water was included for every nine seawater samples to monitor possible contamination. Following centrifugation the supernatant was discarded and DNA was extracted from the pellet using the Qiagen DNeasy (Qiagen DNeasy, Valencia, CA, USA) kit following the manufacturer's guidelines and eluted in 100 µl of buffer. The ethanol wash step of the extraction process is expected to remove most of the Na+ monovalent ions and therefore reduce PCR inhibition due to salinity. DNA was also extracted from the epidermis samples using the Qiagen DNeasy kit.

The PrimerBlast software (NCBI, http://www.ncbi.nlm.nih.gov/tools/primer-blast/) was used to design primers unique to the harbor porpoise that would result in amplicons ranging between 60–80 bp in size, based on records in GenBank. The primers 5′-CGCCCCATCAACACAAAGGTTTGG-3′ and 5′-ACTGGGATGCGGATGCTTGC-3′ flanked the region corresponding to sites 82–119 of the harbor porpoise mitochondrial genome (GenBank: AJ554063; [24]) and resulted in a 62 bp amplicon of the 12S region of the mitochondrial genome. This 38 bp intra-primer sequence was monomorphic in all four of the F&B harbor porpoise, as well as an additional four North Sea and Baltic harbor porpoises from the Natural History Museum of Denmark's tissue archive. Furthermore, the sequence differs by at least one base pair (either a C-A transversion or C-T transition), from the next most similar sequence present in GenBank, which was shared by several cetacean species, none of which are likely to occur at our study sites [25], and differed at 6 nucleotide sites from humans, and harbor and grey seals, which are the only other common marine mammals in the area, and thus represent possible sources of mammalian DNA in the water. To increase sensitivity and specificity of porpoise detection we performed TaqMan qPCRs detection assays. A TaqMan probe specific for the target sequence was designed (5′-TCCTGGCCTTTCTATTAGTTCTTAGCA-3′), modified with 6-Fam dye at the 5′ end and a BHQ1 quencher at the 3′ end.

Taqman qPCRs were performed on a Stratagene Mx3000P and each 25 µl reaction contained 10 µl of DNA extract, 1× PCR buffer, 2.5 mM MgCl2, 1 mM of each primer, 0.1 mM mixed dNTPs, 2.5 µM of probe and 0.2 µl AmpliTaq Gold enzyme (Applied Biosystems) under thermocycling 50°C for 5 min and 95°C for 10 min, followed by 55 cycles of 95°C for 30 sec and 56°C for 1 min. To guard against the incorporation of erroneous data derived from contamination, and to investigate the stochasticity of successful amplifications, the PCR amplification was replicated three times for each sample. One PCR blank (containing ddH_2_O instead of sample) was included for every five PCRs to further monitor for contamination during PCR set up.

For initial investigation we pooled DNA extracts: DNA extracts from the five samples collected inside the F&B pen were pooled, DNA extracts from the five samples collected at less than 10 meters from the pen were pooled, and DNA extracts from thirty-eight samples collected at distances greater than 10 meters from the pen were pooled. Only if DNA was successfully amplified from at least one of the triplicate PCRs of the pooled extracts were the individual extracts included in subsequent PCRs. The amplified PCR products were purified using a Qiagen MinElute PCR purification kit. The species origin of positive PCRs were validated as authentic by cloning using the Topo TA cloning kit (Invitrogen), followed by purification and sequencing of the inserted PCR fragment (Macrogen, Europe). Additional PCRs of 43 bp of the cytochrome b gene (fwd primer: 5′-ACACACCCACTAATAAAAAT-3′; rev primer: 5′-AGCCAAAATTTCATCATGAGGA-3′) and the 53 bp of the hypervariable region of the d-loop (fwd primer: 5′-ACACATACCAATATCTAGTCTTTCCTT-3′; rev primer: 5′-CGGGCTTTAACTTATCGTATGG-3′) using the conditions above were conducted to investigate species identity in one sample, which did not match the porpoise reference sequence.

## Results

**Figure 2:**
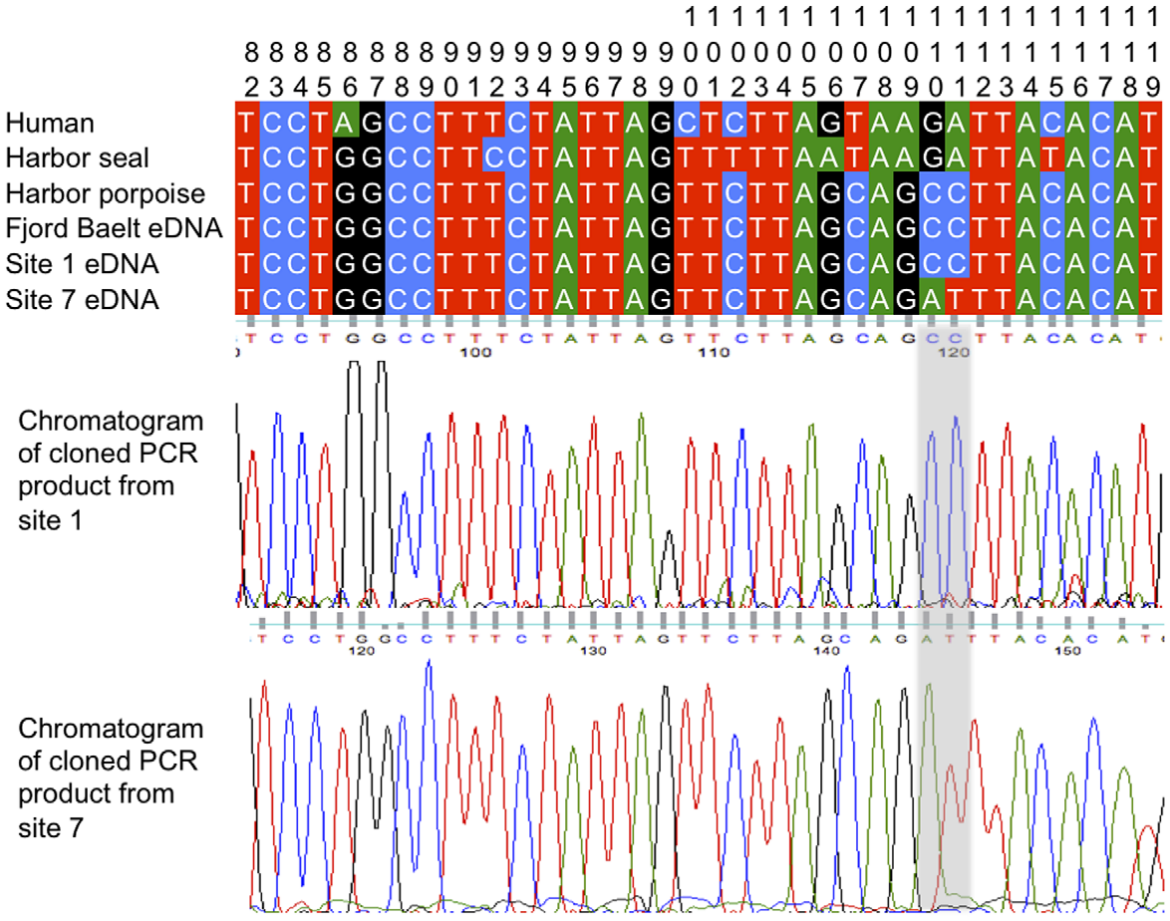
Chromatograms of 12S region mtDNA sequences amplified from two locations in the Baltic aligned to reference sequences. Sites are numbered 82-119 corresponding with the reference harbor porpoise mitochondrial genome (GenBank: AJ554063; [24]).

Porpoise DNA was successfully amplified from a pooled sample of the 5 DNA extracts from each of the 15 ml seawater samples collected from the Fjord&Bælt sea pen (Table 1). The cloned sequences were a 100% match with the reference sequence from GenBank and the sequences generated from skin samples of the four harbor porpoise in the Fjord&Bælt sea pen and the four wild porpoise (Fig. 2). The number of qPCR cycles required for detection was also consistently between 34–35 cycles for all three eDNA PCR replicates, as oppose to 18 for positive controls amplifying tissue-derived DNA (Table 1). Assuming optimal PCR efficiency, this suggests a minimal difference of 4–5 orders of magnitude in DNA concentration, as expected for eDNA extracts generated from such dilute environmental samples. Additionally, porpoise DNA was successfully amplified in all triplicates on individual 15 ml samples. Harbor porpoise eDNA was amplified in one out of three qPCRs on a pooled DNA extract from 5×15 ml samples collected at a distance of less than 10 m from the pen. Beyond 10 m from the pen, we were unable to detect porpoise eDNA (Table 1).

The porpoise genetic detection rate from natural field sites within the western Baltic was validated by comparing with detections of harbor porpoise echolocation clicks detected by the C-PODs. Harbor porpoises were only genetically detected by eDNA at the site (site 1) with the highest percentage (94%) of days that porpoises were acoustically detected by the C-PODs (Table 1). Cloned sequences of qPCR products were a 100% match for the GenBank reference sequence (Fig. 2). Long-term acoustic detection rates at the remaining sampled sites suggest that the lack of genetic detections at some of these sites were false negatives (Table 1). However, it was not possible to confirm this as the C-PODs ran out of battery charge 3–4 weeks before the seawater samples were collected and eDNA typically persists for less than one week (based on studies in freshwater [11], [14] and seawater [26]), and extracellular eDNA can have a turnover time of only a few hours in seawater [27]. Thus it is not known if porpoises were present in the area in the hours prior to sampling.

Environmental DNA was amplified at a site with no acoustic detections of porpoises (Site 7, Table 1). Cloned sequences from the two successful PCRs differed by 2 base pairs from harbor porpoise (Fig. 2), and additional PCRs using primers to target variable regions of the mtDNA *cytochrome b* gene and d-loop confirmed the detection as long-finned pilot whale (*Globicephala melas*) (Table 2), a species occasionally sighted in the Baltic [25].

**Table 2:** Summary of additional PCRs carried out on seawater samples from site 7.

Target gene	cloned PCR product sequence (excluding primers)	GenBank Blast results
*cytochrome b*	CATCAATGACACATTCATTGACCTACCCACTCCATCTAACATC	100% *G. melas*
D-loop	CTTATAAATATATATATATACATGCTATGTATTACTGTGCATTCATTTATTTT	100% *G. melas*

Both primer sets produced a sequence with 100% query coverage and 100% maximum identity match with long-finned pilot whale *Globicephala melas* and no other species.

## Discussion

Our study indicates that species detection of marine mammals using eDNA in seawater samples is possible and thus could have potential use in future genetic monitoring programs. However, it has to be acknowledged that the rate of successful detections in this study was less than found in previous studies using a comparable protocol to sample freshwater ecosystems (e.g. [9], [11]). There may be several reasons for this including those identified above, for example, the greater dispersal and dilution of eDNA in marine ecosystems compared to lakes and ponds. Porpoise eDNA was successfully amplified from five different 15 ml water samples collected at different points around the perimeter of the Fjord&Bælt sea pen and therefore appears to be relatively homogenously distributed throughout the 4 million liter sea pen. This is consistent with findings from studies on pond water [9], [11], and demonstrates that species detection of marine mammals at high density is possible using eDNA from seawater samples. However, these controlled conditions differ in several respects from natural conditions. In particular, the animal density in the sea pen is higher than in most natural populations. Furthermore, the sheltered pen is less subject to wave and wind action and eDNA from porpoises within the pen may not be dispersed to as great an extent as under natural conditions and is thus more concentrated here. Comparing the number of qPCR cycles required for detection at the natural site (site 1) with the sea pen would suggest a minimal difference of 4–5 orders of magnitude in DNA concentration. Increasing the sample volume would be expected to reduce the rate of false negatives, if the DNA could be concentrated (*e.g.*, [26]). Thomsen *et al.*, [26] were able to genetically detect several fish species by passing half a liter of a 1.5 liter pool of 30 ml Baltic seawater samples through a nylon filter and then extracting the DNA collected from the filter. Such an approach may improve the detection rate and the applicability of this approach to species detection using seawater samples. The detection probability is likely to be dependent upon density of the target species, the amount of DNA released by the organism through excretory processes, and the amount of degradation by local environmental factors such as endogenous nucleases, hydrolysis, UV radiation and bacterial action [14], it is therefore likely to be vary greatly amongst target species and study locations.

Despite these limitations, the successful genetic detection of harbor porpoise at one location where the species was also acoustically detected indicates that, with optimization, genetic detection of marine mammals could provide a useful non-invasive genetic monitoring tool that could compliment visual and acoustic surveys. The genetic detection of long-finned pilot whale from seawater collected at our most easterly study site, about 75 km southeast of Bornholm, highlights the potential for this method to detect species that are rare visitors to an area. No other genetic work on this species has been conducted in the laboratories in which the work was undertaken, which, in addition to the rigorous clean lab procedures employed, makes laboratory contamination an unlikely source of the DNA. Long-finned pilot whales are infrequently sighted in the Baltic, however, there were two potential but unconfirmed sightings of pilot whales in the western Baltic in July 2011 (www.hvaler.dk). The genetic detection presented here remains the only confirmed detection of this species in the Baltic during this period. C-PODs can also detect clicks in the frequency range produced by pilot whales. However, the C-POD file contained no such click detections. As acoustic monitoring ended one month prior to eDNA water sampling, the acoustic data cannot act to validate the presence of pilot whales contemporaneously with eDNA sampling at this site. Pilot whales are much larger than harbor porpoise and are typically more gregarious, and they would therefore be expected to excrete and shed more eDNA and be more easily detectable using this method. However, the indirect method of sampling DNA used here means that it is not possible to determine whether the animals were recently in the area, or whether the eDNA originated elsewhere and travelled with sea currents, or originated from the remains of a long dead animal (e.g. DNA from mammoths has been successfully amplified from riverine sediment samples [28]).

Two lines of further investigation could be used to clarify these possible scenarios. Firstly, determining if the detected eDNA is cellular, (for example contained within slough skin cells or fecal material), or extracellular DNA by using extraction methods designed to isolate extracellular DNA from DNA extracted by cell lysis (e.g. [29]). As extracellular DNA degrades much faster (hours) than cellular DNA (days) [27], it would be more likely to have originated from a living animal recently in the area than have travelled on currents or be from remains of dead specimens. Secondly, the controlled release of DNA of a non-native species of known concentration could be used to experimentally investigate the longevity of DNA in the water column and its propagation by wind, wave and ocean currents.

Our results suggest that, as expected, species detection using eDNA is less reliable in the marine ecosystem than in freshwater ecosystems. However, our results do suggest that marine mammal detection by amplifying eDNA from seawater samples and using short species-specific DNA sequences as DNA barcodes [30] is possible. However, it is likely to be dependent upon the size, behavior and density of the target species. The volumes of seawater sampled in this study were small to allow comparison with previous freshwater studies [9], [11], which sampled similar volumes of water. With optimization and larger volumes of seawater this method could have potential to compliment current visual and acoustic methods of species detection of marine mammals and provide a low-cost, logistically simple method of obtaining basic genetic data such as species presence or even intraspecific variation in short diagnostic fragments.
